# A Linear Ultrasonic Motor Based on Coupling Vibration Mode

**DOI:** 10.3390/mi13111852

**Published:** 2022-10-28

**Authors:** Danhong Lu, Hong Liu, Jianqiao Xu, Ting Yang, Hanwen Hu

**Affiliations:** 1 School of Electric Power Engineering, Nanjing Institute of Technology, Nanjing 211167, China; 2 Jiangsu Electric Power Company Xiangshui County Power Supply Company, Lianyungang 224600, China

**Keywords:** linear ultrasonic motor, coupling vibration mode, eccentric constraint, output characteristics

## Abstract

A coupled linear ultrasonic motor (LUSM) based on an eccentric constraint was proposed. Two pieces of oblique piezoelectric ceramics were arranged at each end of the elastomer, and the polarization direction of the ceramics was vertically upward. Using the tilting characteristics of the piezoelectric ceramics, the two ends of the fixed piezoelectric ceramics formed an eccentric restraint on the motor, providing conditions for the motor to generate coupled modes. When the elastomer of the motor generated the coupling vibration, the motion trajectories of the driving feet ends were oblique straight lines, and the oblique straight-line motion trajectories of the upper and lower driving feet ends were in opposite directions, driving the upper and lower sliders to run simultaneously. The stator parameters were optimized by using ANSYS to obtain larger amplitudes for the ends of the driving feet in both X and Z directions. The structure and operation principle of the motor are explained in detail. A prototype was fabricated to study the arrangement scheme with fixed constraints at the ends of the motor. The frequency–velocity characteristics, voltage–velocity characteristics, and mechanical characteristics of the motor were tested. The no-load speed and maximum output power were measured to be 45.9 mm/s and 3.24 mW.

## 1. Introduction

Ultrasonic motors (USMs) have many merits, such as working in a wide velocity range from 1 nm/s to 1 m/s and a large thrust force with simple construction, quick response, no electromagnetic radiation [1], higher position accuracy [2,3], and being self-locking when the power is off [4,5]. With these advantages, USMs have been widely applied in many fields such as medical robots [6,7], digital cameras, aerospace equipment, etc. [8,9].

Compared to electromagnetic linear motors, linear ultrasonic motors have a simple structure, fewer parts [10], and no longitudinal edge effect on the driving force, and their efficiency is higher in small-volume applications. Therefore, in recent years, linear ultrasonic motors have received widespread attention.

As a kind of USM, linear ultrasonic motors can be divided into single-mode LUSMs [11,12,13] and multi-mode LUSMs [14]. For multi-mode LUSMs [15,16], they use the hybrid of at least two modes to achieve the driving purpose. Multi-modal motors offer a high degree of design flexibility and are available in a variety of structural forms, including frog, rectangular, cylindrical [17,18], and other types [19,20,21]. For linear ultrasonic motors, multi-modal motors can generally be divided into composite [22] and coupled modes.

B. Delibas et al. proposed a first longitudinal vibration with a second bending vibration (L1-B2)-type composite modal piezoelectric ultrasonic motor [23,24]. The dimensions of the vibrator were set as 16 mm × 7 mm × 4 mm (length × width × thickness). The motor excited the modal component of the first longitudinal vibration by applying two sinusoidal signals with a phase difference of 180 degrees. The motor had a good controllability and a wide range of moving speeds, from 1.0 × 10^−6^ m/s up to 1.0 m/s. The modal component of the second bending vibration in the motor interfered with the modal component of the first longitudinal vibration.

W. Chen et al. proposed a higher-order composite-mode ultrasonic motor, which can be seen as a bolted-tight transducer [25]. Different voltages were applied to the ceramic in order to simultaneously obtain a second longitudinal vibration component and a fifth bending vibration component in the motor. The bending PZT ceramic, which was divided into two parts, was excited by different voltages, resulting in alternating tensile vibrations in both parts of the ceramic, which was the key to the motor’s ability to generate both longitudinal and bending vibration components in a similar frequency range. The maximum no-load speed and thrust of the prototype at 450 Vp-p were 439 mm/s and 37 N, respectively. Gal Peled et al. used multiple rectangular piezoelectric rods polarized along the thickness direction to simultaneously excite the simultaneous generation of first longitudinal and second bending resonant modes for motor design [26]. The no-load speed and thrust of each of its driving units could reach 300 mm/s and 4 N.

Composite modal motors usually require two sets of excitation power supplies to excite the two modes of the motor simultaneously, and the resonant frequencies of the two different modes need to be adjusted so that they are as close as possible to each other. In the past year, coupled-mode LUSMs have been developed. In these systems, the operational modes include two modal components that are coupled together so that when one oscillates, the other oscillates simultaneously. Therefore, just one set of power supplies is often needed for excitation in this coupled LUSM.

Liu et al. proposed and tested a bi-directional standing wave linear piezoelectric actuator [27]. The working mode of the piezoelectric actuator was a special coupled mode that contained both longitudinal and bending (L-B) vibration components. The piezoelectric actuator contained two transducers to push the runner forward and backward, respectively. The unsymmetrical boundary condition was the key factor for the generation of this coupling vibration. With a voltage of 400 Vp-p, the maximum no-load velocity and thrust force of the prototype could achieve 244 mm/s and 9.8 N.

Liang et al. proposed a novel coupled-mode linear piezoelectric ultrasonic motor [28]. The proposed motor used first-order longitudinal vibration to stimulate the asymmetric structure of the stator to produce oblique vibration on the driving feet. Two pieces of PZT plates were bonded on the upper and lower surface of the elastomer and the driving parts were arranged at two ends. With a size of 62 mm × 16 mm × 16 mm, the maximum no-load velocity and thrust force of the prototype could achieve 127.31 mm/s and 2.8 N under the voltage of 150 Vp-p and preload of 40 N.

As can be observed from the various motors already described above, coupled-mode ultrasonic motors are easier to construct and have less interference when choosing a frequency and setting up a driving scheme.

The excitation ceramic is generally at the surface, and the driving foot is generally located at the end of coupled-mode linear motor; the application of the preload is relatively simple and allows for a large thrust force. However, the ceramics are arranged on the upper and lower surfaces of the elastomer, and the thickness of the motor is generally high.

Different from the motors proposed by Liu, Liang, et al., the LUSM in this paper contains two PZT ceramics at two ends of the elastomer so that the driving parts can be arranged at the upper and lower surfaces to push the sliders to move forward and backward. The stator is fixed and excited by the bronze electrodes. The working mode is a special coupling vibration mode that contains both longitudinal and bending components. The coupling vibration is generated by the unsymmetrical boundary condition. The cross-sectional area of PZT ceramics is designed to be parallelogram shapes. The polarization of PZT ceramics is in the positive *Z*-axis direction. When the AC voltage is applied to PZT ceramics, the d15 and d33 modes occur simultaneously. According to the arrangement schemes of the PZT ceramics, a motor based on coupling vibration mode has been proposed: an LUSM-based first-order longitudinal vibration and second-order bending vibration (L1-B2)-coupled vibration mode. The structure of the motor is designed, and its working principle is illustrated. The vibration mode of PZT ceramics is clarified in detail. Finite element method simulation allows us to obtain the results of harmonic response analysis and transient analysis of the system and to verify the feasibility of the model. The motors are fabricated, and their vibration characteristics and output performance are investigated through experiments.

## 2. Motor Structure

The linear ultrasonic motor based on the coupling vibration mode proposed in this paper is mainly composed of a stator, sliders, and bronze electrodes. The stator includes two PZT ceramics, a metal elastomer, and two driving feet. PZT ceramics are pasted at two ends of the elastomer, and the driving feet are arranged on the upper and lower surfaces of the elastomer to drive the sliders. The left and right ends of the stator are fixed by bronze electrodes to energize the PZT ceramics.

The cross-section of PZT ceramics is a parallelogram, and it is polarized in the *Z*-axis direction. An isotropic eccentric constraint is applied to the motor by the oblique piezoelectric ceramic, which causes a coupled L1-B2 vibration mode to the elastomer. In reaction to the oblique electric field, the oblique piezoelectric ceramic produces both longitudinal and bending vibrations. The longitudinal vibration of the ceramic excites the longitudinal vibration component of the L1-B2-coupled mode, and the bending vibration of the ceramic excites the bending vibration component of the L1-B2-coupled mode, resulting in an oblique linear motion of the driving foot to drive the load.

**Figure 1:**
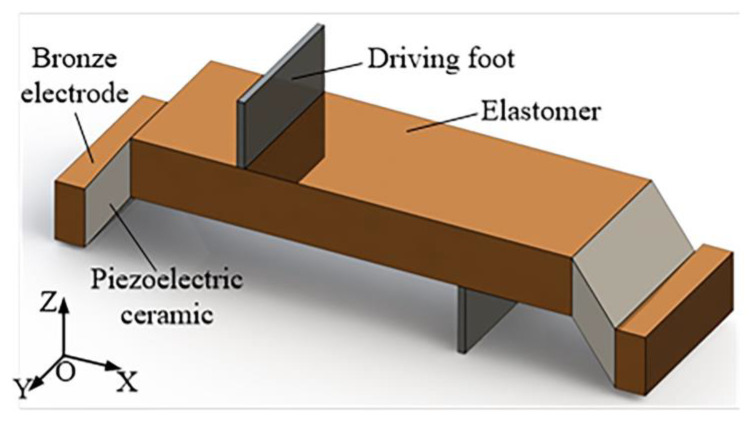
The structure of motors: the motor works on L1B2 coupling.

According to the arrangement schemes of the PZT ceramic, the following motor is proposed: the motor works on the coupling vibration mode of first-order longitudinal and second-order bending, as shown in Figure 1.

## 3. Operating Principle

### 3.1. Vibration Mode of PZT Ceramic

**Figure 2:**
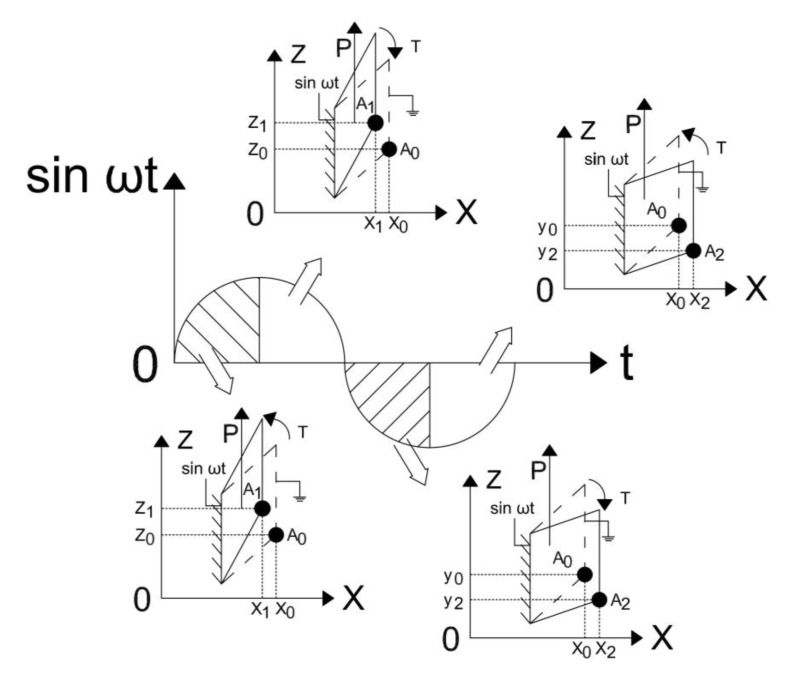
Vibration principle of the PZT ceramic. (P and the arrow next to it mean the direction of polarization of the ceramic. T and the arrow next to it mean the direction of motion of the ceramic).

PZT ceramics in the motor are pasted at two ends of the stator. Take one vibration period of a piece of PZT ceramic as an example, and its vibration deformation is shown in Figure 2.

The dotted lines represent the undeformed shape of the PZT ceramic, and the solid lines represent the deformed shape. The left side of the PZT ceramic is connected to the bronze electrode, which provides power and holds it in place. The right side of the PZT ceramic is connected to the elastomer and transmits the vibration to it.

From t = 0 to t = π, the PZT ceramic contracts in the direction of the *X*-axis and twists in the positive direction of the *Z*-axis. When t ∈ (0, π/2), the lower right endpoint of the PZT ceramic moves from point A0 to point A1 (x0–x1, z0–z1); when t ∈ (π/2, π), the lower right endpoint of the PZT ceramic moves from point A1 to point A0 (x1–x0, z1–z0).

From t = π to t = 2π, the PZT ceramic stretches in the direction of the *X*-axis and twists in the negative direction of the *Z*-axis. When t ∈ (π–3π/2), the lower right endpoint of the PZT ceramic moves from point A0 to point A2 (x0–x2, z0–z2); when t ∈ (3π/2–2π), the lower right endpoint of the PZT ceramic moves from point A2 to point A0 (x2–x0, z2–z0).

### 3.2. Operating Principle of the Motor

**Figure 3:**
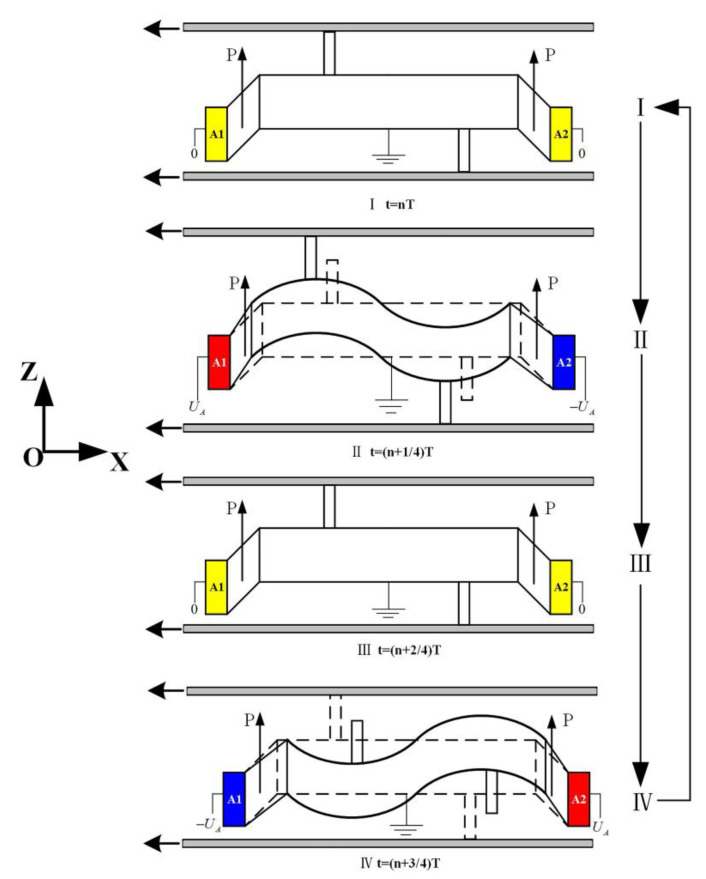
Operating principle of the motor. (P and the arrow next to it mean the direction of polarization of the ceramic.)

In one vibration period, the motor that works on the L1-B2 coupling vibration mode is operated as shown in Figure 3.

The first quarter: the motor runs from state I to state II. During this process, the upper driving foot moves from the initial position to the leftmost position along the negative *X*-axis and to the highest position along the positive *Z*-axis. The lower driving foot moves from the initial position to the leftmost position along the negative *X*-axis and to the lowest position along the negative *Z*-axis.

The second quarter: the motor runs from state II to state III. During this process, both the upper and lower driving feet return to their initial positions from the positions they moved to in the second quarter.

The third quarter: the motor moves from state III to state IV. During this process, the upper driving foot moves from its initial position to the rightmost position along the positive *X*-axis and to the lowest position along the negative *Z*-axis. The lower driving foot moves from the initial position to the rightmost position along the negative *X*-axis and to the highest position along the positive *Z*-axis. The driving foot gradually moves from the contact state to the separated state with the slider.

The fourth quarter: the motor runs from state IV to state I. During this process, both the upper and lower driving feet return to their initial positions from the positions they moved to in the third quarter.

The operation sequence of I-II-III-IV-I enables the stator to drive the upper slider and lower slider to move along the negative *X*-axis.

## 4. FEM Analysis

### 4.1. Modal Analysis

APDL code was used in ANSYS to simulate the stator’s modal vibration. The material of the elastomer was phosphor bronze with a mass density of 8624 kg/m^3^, elastic modulus of 101 × 10^9^ pa, and Poisson’s ratio of 0.373. The material of the driving feet was 45# steel with a mass density of 7800 kg/m^3^, an elastic modulus of 206 × 10^9^ pa, and a Poisson’s ratio of 0.28. PZT-5 piezoelectric ceramics using the d15 and d33 modes were selected as the piezoelectric components, whose physical parameters are:

(1) $$d=\left[\begin{array}{cccccc} 0 & 0 & 0 & 0 & 10.5 & 0\\ 0 & 0 & 0 & 10.5 & 0 & 0\\ -4.1 & -4.1 & 14.1 & 0 & 0 & 0 \end{array}\right]\times 10^{-10}\;C/N$$

(2) $$C^{E}=\left[\begin{array}{cccccc} 13.2 & 7.1 & 7.3 & 0 & 0 & 0\\ 13.2 & 7.3 & 7.3 & 0 & 0 & 0\\ 7.3 & 7.3 & 11.5 & 0 & 0 & 0\\ 0 & 0 & 0 & 3 & 0 & 0\\ 0 & 0 & 0 & 0 & 2.6 & 0\\ 0 & 0 & 0 & 0 & 0 & 2.6 \end{array}\right]\times 10^{10}\;N/m^{2}$$

(3) $$\varepsilon^{T}=\left[\begin{array}{ccc} 1.3 & 0 & 0\\ 0 & 1.3 & 0\\ 0 & 0 & 1 \end{array}\right]\times 10^{-9}\;F/m$$

where Equation (1) is the piezoelectric stress matrix, Equation (2) is the elastic coefficient matrix, and Equation (3) is the permitting matrix at constant stress.

**Figure 4:**
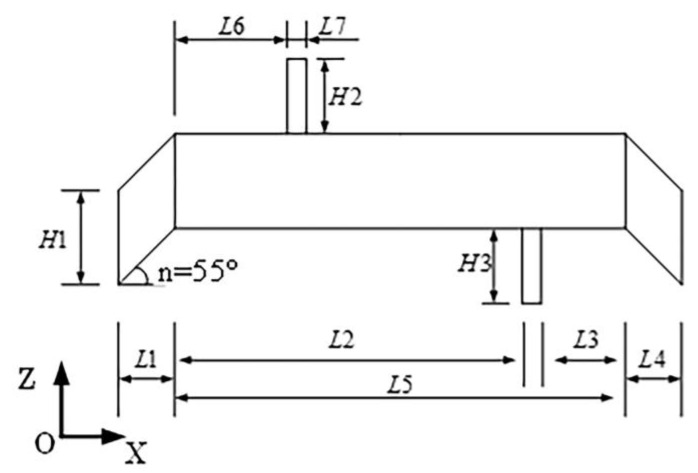
Parameters of the stator.

During the simulation, the SOLID45 element was employed to mesh the elastomer and driving feet and the SOLID5 element was employed to mesh the PZT ceramics. The finite element model of the stator was founded according to the parameters in Figure 4 and Table 1. The dimensions of both the motor and the ceramic in the *y*-axis direction were 10 mm. 

**Figure 5:**
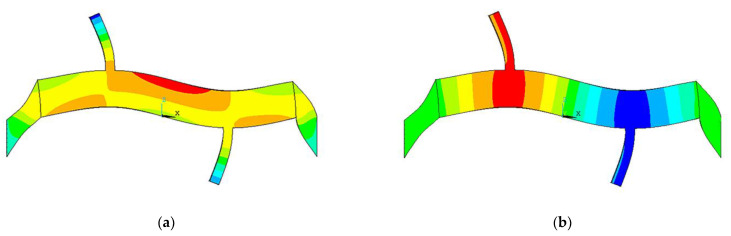
Modal analysis: (**a**) X-directional vibration mode; (**b**) Z-directional vibration mode.

The vibration mode of the stator was extracted by modal analysis. Under ideal end-fixation conditions, the extracted vibration modes in the x and z directions are shown in Figure 5, where the end-fixation constraint would be applied directly to the outer end of the ceramic. The mode would be the L1-B2 coupling mode. The natural frequency of the motor would be analyzed to be 49.098 kHz by ANSYS.

**Figure 6:**
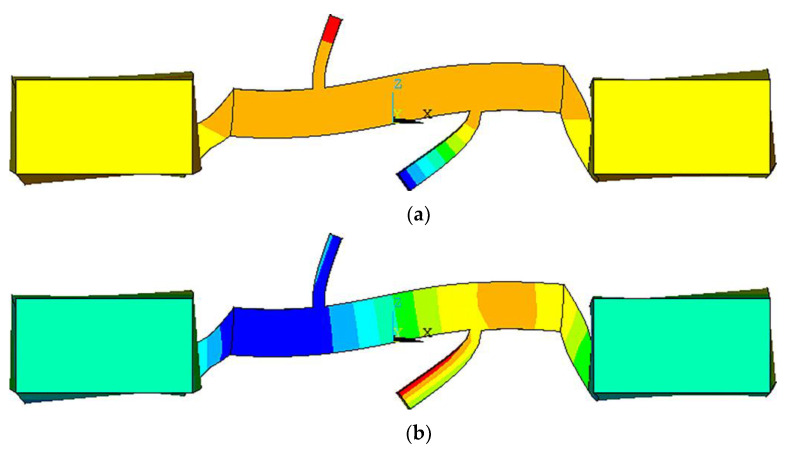
Model analysis: (**a**) X-directional vibration mode with aluminum bars for ends fixing; (**b**) Z-directional vibration mode with aluminum bars for ends fixing.

However, in the actual experimental process, this ideal end constraint condition was hard to achieve directly. In this paper, a large mass aluminum bar was attached to the outer end of the ceramic, and the ends of the aluminum bars were fixed to obtain the end constraints. Modal analysis showed that it still had the L1-B2 coupling mode shown in Figure 6, but the modal frequency changed to 24.007 kHz.

### 4.2. Harmonic Response Analysis

Harmonic response analysis was performed around the intrinsic vibration frequency to determine the exact operating frequency of the coupled motor and study its frequency response under periodic voltage loads. 

A high-frequency sinusoidal alternating current (AC) of 100 V was applied to the left-sided oblique piezoelectric ceramic, and a high-frequency sinusoidal AC of −100 V was applied to the right-sided oblique piezoelectric ceramic to excite the desired longitudinal–bending-coupled vibration mode of the motor under the condition of surface restraint. The harmonic response analysis method of the complete solution method was used, and the frequency analysis ranged from 23.5 kHz to 24.5 kHz, with a sub-step of 20 Hz and a motor structural damping ratio of 0.02.

**Figure 7:**
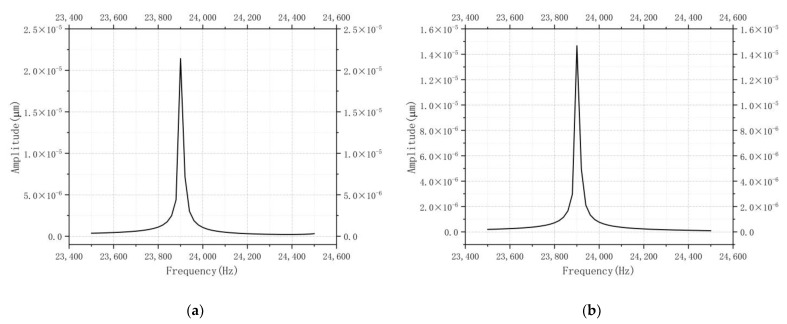
Frequency–amplitude curves: (**a**) X-direction harmonic response analysis; (**b**) Z-direction harmonic response analysis.

The top points of the driving feet on the upper surface were selected as the observation points of the harmonic response analysis, and the obtained amplitude–frequency relationships are shown in Figure 7. When compared, the X and the Z directions basically overlapped and had equal resonant frequencies. From the observation of the analysis results, it can be seen that when the frequency was 23.9 kHz, both the two directional curves and the amplitude–frequency response curves of the driving feet in the X directional vibration amplitudes of the motor-driving feet were extremely large. It was determined that the excitation scheme for the motor to produce the demand coupling mode was 23.9 kHz.

### 4.3. Transient Analysis

In order to obtain the trajectory of the driving foot, it was verified that the motor made an oblique linear motion. The same excitation scheme was used for the harmonic response analysis. The transient solution was performed by applying asymptotically varying same-frequency reverse voltage loads to the two ends of the oblique piezoelectric ceramic with several periods of 800 and a number of sub-steps of 8.

**Figure 8:**
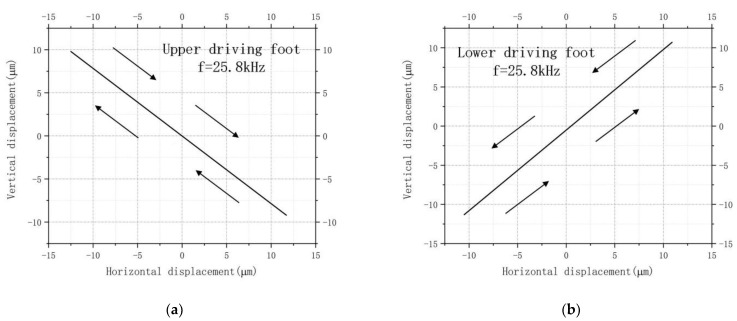
Trajectories of the driving feet: (**a**) the upper driving foot; (**b**) the lower driving foot.

The trajectories on the driving feet of the motor that worked on the L1-B2 coupling vibration mode are shown in Figure 8. It can be seen that the trajectories of the driving feet were oblique lines. The amplitude of vibration along the *X*-axis determined the output velocity of the motor, and the amplitude of vibration along the *Z*-axis determined the maximum preload that the motor could endure.

As shown in Figure 8, the trajectories of the driving feet movements on both the upper and lower surfaces were oblique straight lines. The arrows were used to indicate the direction of motion. The maximum horizontal displacement of the driving foot on the upper surface was about 12.5 μm, and the vertical displacement was about 9.8 μm. The maximum horizontal displacement of the driving foot on the lower surface was about 10.9 μm, and the vertical displacement was about 11.3 μm.

From the transient analysis results, the motor’s output characteristics were balanced. The inclined linear motion trajectories of the upper and lower surface driving feet ends were tilted in opposite directions, thus enabling the same direction of motion of the double sliders. The analyzed structure was consistent with the initial operating prognosis of the motor.

## 5. Experiment

### 5.1. Prototype Motor

**Figure 9:**
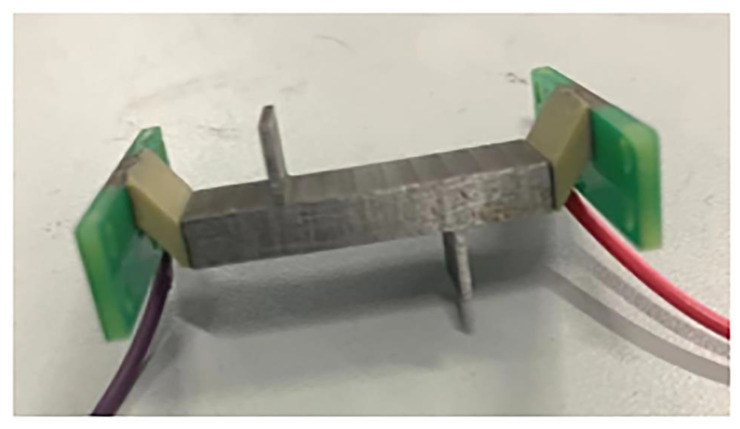
The stator of the motor.

The parameters in Table 1 were chosen to construct the prototype. The linear ultrasonic motor prototype is shown in Figure 9. The stator body of the motor was made entirely of steel, and both the ends and surfaces of the stator were polished smoothly to ensure that the oblique piezoelectric ceramics could be well-arranged at both ends of the stator elastomer according to the above-mentioned scheme, stimulating the required coupling mode. To power the ceramics and produce the end eccentric constraint with the oblique piezoelectric ceramics, light PCB material plates were stacked at both ends of the oblique piezoelectric ceramics.

Due to its own weight being light and thin, a single PCB material board does not have an optimal constraint on the end of the motor during prototype production. The oscillation of the motor’s piezoelectric ceramics would cause resonance between the PCB material board and the motor, resulting in some energy loss. The actuator’s longitudinal bending coupling mode cannot be well excited using this method.

**Figure 10:**
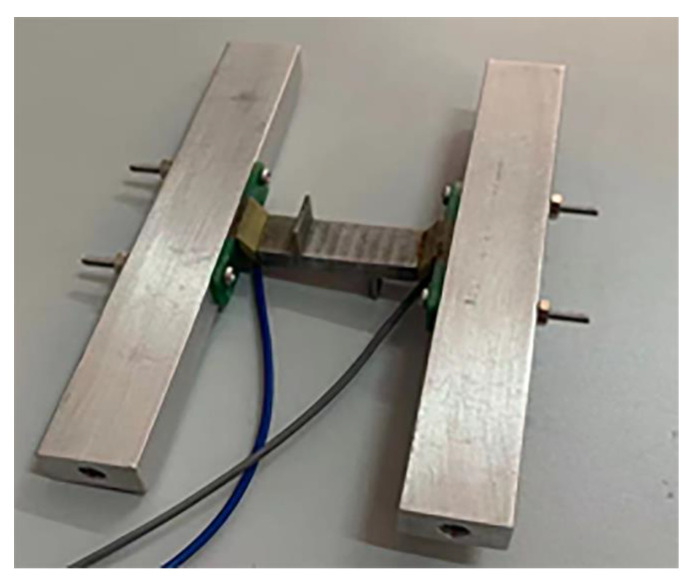
Improved motor structure.

In order to make the end of the motor be in a state of complete boundary constraints to eliminate the energy loss of the end, the fragility of oblique piezoelectric ceramic structures was considered. After several attempts, the motor at both ends were of a lighter quality but with a certain degree of hardness, and the hard aluminum rod was fixed at both ends of the motor as its boundary constraints. The scheme reduced the resonance of the piezoelectric ceramics, and aluminum rods and hard aluminum rods were easily installed. Its structure is shown in Figure 10.

### 5.2. Test Platform

**Figure 11:**
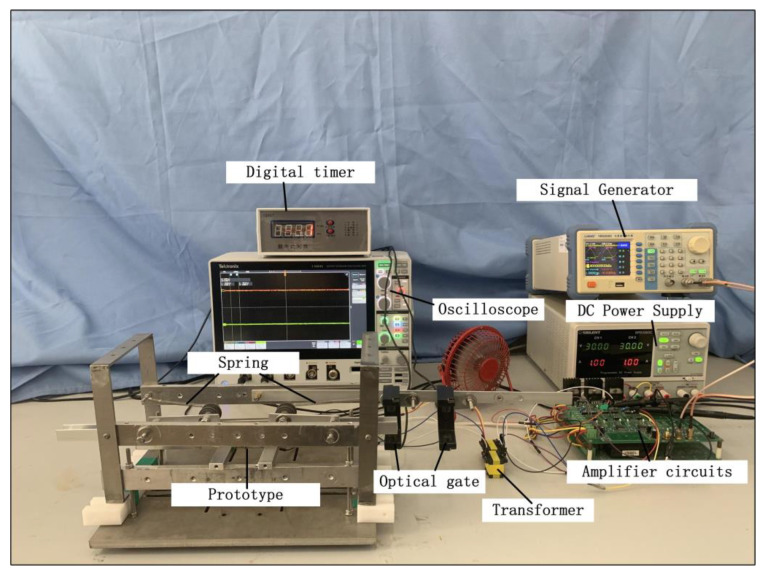
The platform of the experiment.

An ultrasonic motor test rig based on an isotropic eccentric constraint was designed and built, as shown in Figure 11. A DC power supply was used for conversion, and the resulting high-frequency AC voltage was transmitted to an oblique piezoelectric ceramic for excitation. After a PCB board was closely bonded to the piezoelectric ceramic on both sides of the motor, the motor was fixed to the platform using a rigid aluminum bar to realize the fixation of both ends of the motor. Then, the motor was mounted and fixed to the platform using a solid aluminum bar with high hardness. The sliders and driving feet were in full contact without any gap. An oscilloscope was used to observe the phase difference of the AC voltage applied on both sides of the motor. The driving speed of the motor was measured by recording the time of the kinematic passing through the photogate. On the experimental platform, the end oblique piezoelectric ceramic was used for fixation and excitation, and there was only one driving foot on each of the upper and lower surfaces. Due to the strength of the oblique ceramic, the bonding of the ceramic to the elastomer, the end fixation of the ceramic, the horizontal balance of the slider, and other process reasons, preload was applied in the prototype using gravity with a preload of approximately 1.3 N.

### 5.3. Impedance Analysis

Due to the dielectric properties of piezoelectric ceramics, ultrasonic motors are generally externally capacitive under the operating frequency band, so the impedance characteristics test was beneficial to the impedance matching of the motor and power amplifier circuit, thus improving the output characteristics of the motor.

**Figure 12:**
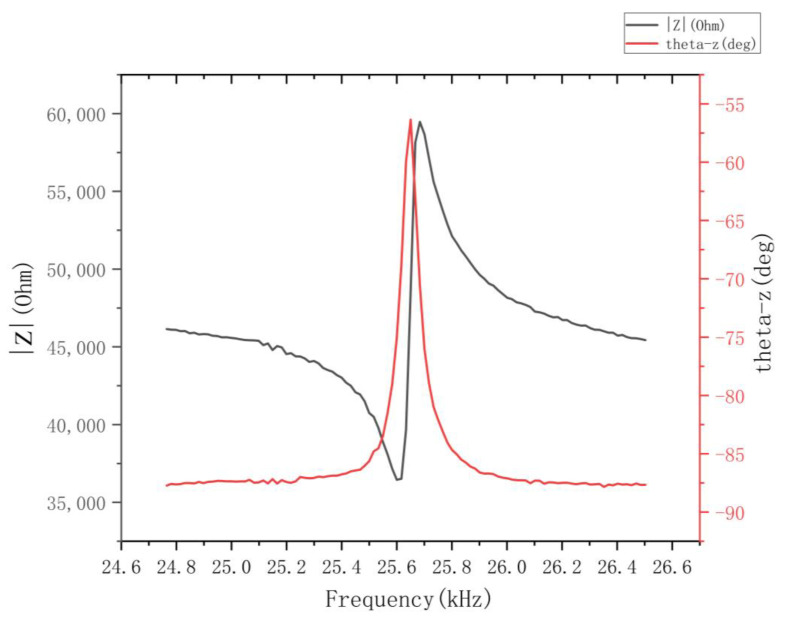
Stator impedance characteristic curve.

The impedance analyzer was used to test the impedance characteristics of the stator, and the frequency range of the scan was selected to be 20 kHz to 30 kHz; the impedance characteristics of the stator were obtained, as shown in Figure 12. They were end-bound by means of aluminum bars.

As shown in Figure 12, the stator impedance amplitude reached a maximum at a frequency of 25.684 kHz; the impedance phase reached a maximum at 25.64 kHz. There was a discrepancy between the modal frequencies measured by the impedance analysis experiments and the frequencies analyzed by the finite element analysis. Due to the actual test, there was a certain error in the fabrication and processing of the stator, and when adding ceramics and performing ends fixing, it made a sticky layer in the motor structure, which led to a certain error in the sweeping results, but it was still within the test error.

### 5.4. The Relationship between Frequency and Velocity

**Figure 13:**
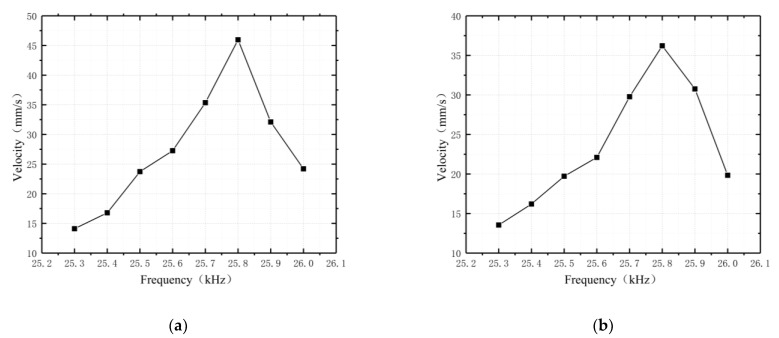
Velocity versus frequency characteristics: (**a**) upper surface driving foot; (**b**) lower surface driving foot.

The ultrasonic motor used in this paper was excited by the high-frequency alternating current, with a 180° phase difference between the two sides of the voltage, so changing the input AC voltage frequency would affect the polarization of the piezoelectric ceramic. After the prototype was made, the operating frequency of the motor and the simulation were inaccurate due to the manufacturing conditions and process, so the voltage V = 600 V, and the driving speeds of the two driving feet on the upper and lower surfaces were studied at different frequencies to verify the working performance of the motor. The test results are shown in Figure 13.

According to the test results, the motor achieved the best operating performance at 25.8 kHz of frequency. The maximum driving speed of the motor was 45.9 mm/s at the upper surface driving foot and 36.2 mm/s at the lower surface driving foot.

### 5.5. The Relationship between Voltage and Velocity

**Figure 14:**
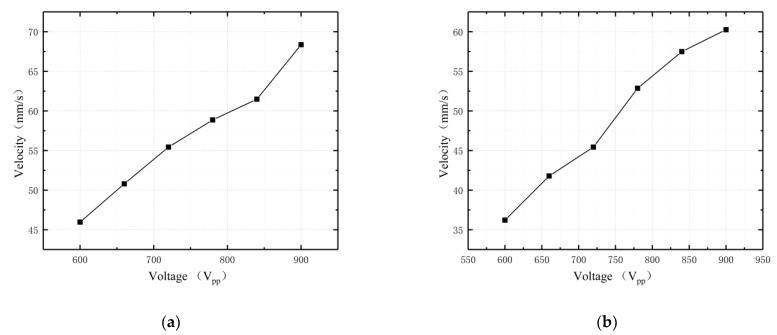
Velocity versus voltage characteristics: (**a**) upper surface driving foot; (**b**) lower surface driving foot.

Based on the inverse piezoelectric characteristics of oblique piezoelectric ceramics, it is known that as the excitation voltage rises, the stronger the motor stator vibration and the larger the amplitude. Thus, the driving force applied to the kinematics by the stator driving foot under a certain preload is greater, resulting in a faster motor output kinematic operation. In light of this principle, to achieve the relationship between motor driving speed and voltage amplitude under the same frequency of AC voltage, 25.8 kHz was selected as the operating frequency of the motor, and the upper and lower surface driving feet worked separately. The voltage amplitude multiplier was expanded to measure the motor driving speed. The experimental results of the two driving feet are shown in Figure 14.

In accordance with the experimental results obtained at 25.8 kHz, with the increase in voltage, the output speed of the motor increased continuously. The driving speeds of the feet reached the maximum of 68.36 mm/s and 60.25 mm/s, respectively, when the upper and lower surface driving feet worked separately.

### 5.6. Mechanical Characteristics

**Figure 15:**
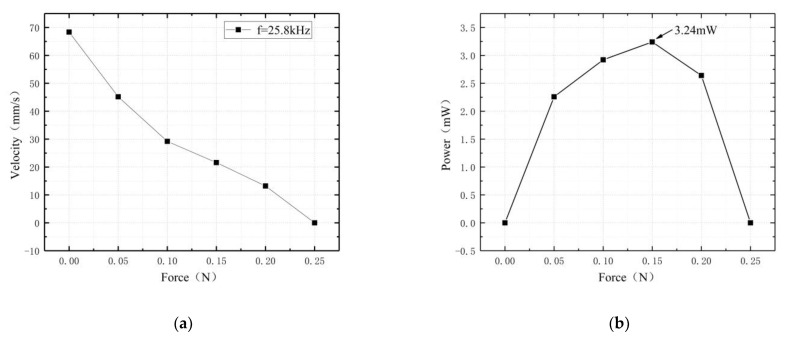
Mechanical characteristics: (**a**) the relationship between force and velocity; (**b**) the relationship between force and power.

To test the mechanical carrying characteristics of the coupled motor, 25.8 kHz was selected as the operating frequency of the motor, by means of weights and roller construction to apply the load. To measure the optimal carrying capacity of the motor, V = 900 V, and the upper surface driving foot was taken as the research object to measure the mechanical carrying performance of the motor; the experimental results are shown in Figure 15.

According to the experimental results, the driving speed of the motor decreased gradually to 0 at 25.8 kHz as the applied load increased. The output power of the motor had a peak value, and the maximum power output of the upper surface driving foot was 3.24 mW.

### 5.7. Analysis of Emperiment Results

According to the experimental results, the maximum thrust of the prototype was 0.25 N, the maximum speed was 45.7 mm/s, and the maximum output power was 3.24 mW; the output of the motor was on the small side.

Comparing this motor with the two coupled modal motors [27,28], Table 2 shows the comparison of the amplitudes in the X and Z directions under transient analysis of the three motors. It can be seen that the prototype designed in this paper had greater amplitude in the X and Z directions than the other two motors because the driving feet of the prototype in this paper were located on the upper and lower surfaces of the elastomer, whereas the driving feet of the other two motors were located at the ends; the driving foot of the motor in this paper was also longer.

However, the thrust and speed of the motors in this paper were smaller than those of the other two motors, as shown in Table 3. The reason for this is that the experimental platform of the prototype was not perfect; specifically: (1) there was a gap between the actual situation and the ideal situation, which was not entirely fixed; (2) the slider was not balanced enough, the stability was poor, and the load during the experimental process posed a greater impact on its horizontal stability; (3) the preload was not applied to the ideal value due to the influence of the ceramic structure of the end; (4) the slider used in this paper produced deformation during the experimental process; and (5) the driving foot was too long and thin, which affected the transmission of the motor thrust. The integrated design of the motor test platform, combined with the actual load, would effectively improve the output of this motor in the future.

## 6. Conclusions

In this paper, we proposed an eccentrically constrained, coupled linear ultrasonic motor based on oblique piezoelectric ceramics. The key to this paper was the use of oblique piezoelectric ceramics. Firstly, the oblique piezoelectric ceramics provided isotropic eccentric constraints to give the motor the coupled operating mode of L1-B2, and secondly, the tilted electric field in the oblique ceramics caused the ceramics to vibrate both longitudinally and torsionally, which in turn, excited the coupled-mode vibration pattern of the motor.

Then, the working principle of this motor was elaborated. The finite element model was established, and finite element analysis was performed using ANSYS software. The prototype was designed and fabricated, and its operating characteristics and mechanical characteristics were tested. The experimental results obtained by testing the frequency characteristics, voltage characteristics, and mechanical characteristics of the coupled motor verified that the isotropic eccentric restraint had a facilitating effect on the excitation of the coupled vibration mode and verified the feasibility and correctness of the motor design.

## 7. Patents

Patent title is “Coupled-Mode Ultrasonic Motor Based on Co-axial Eccentricity Constraint and Oblique Piezoelectric Ceramics”. The patent disclosure number is CN112165273A.

## Figures and Tables

**Table 1:** Parameters of the stator (unit: mm).

Parameters	L1	L2	L3	L4	L5	L6	L7	H1	H2	H3
Values	3	20	7	3	28	7	1	4	6	6

**Table 2:** Comparison of the amplitude of the three motors.

Motor	Amplitude Values in the X-Direction (μm)	Amplitude Values in the Z-Direction (μm)
Proposed motor	12.5	11.3
The motor by Liu, Y. et al. [27]	6.4	6.2
The motor by Liang, W. et al. [28]	2.51	3.41

**Table 3:** Comparison of the output performances of the three motors.

Motor	Maximum Velocity (mm/s)	Maximum Thrust (N)
Proposed motor	45.9	0.25
The motor by Liu, Y. et al. [27]	244	9.8
The motor by Liang, W. et al. [28]	127.31	2.8

## Data Availability

Data available upon request due to restrictions e.g., privacy or ethical. The data presented in this study are available upon request from the corresponding author. The data are not publicly available due to privacy.
